# Breaking the limits of experimental pancreas transplantation: Working toward the clinical ideal graft

**DOI:** 10.3389/frtra.2022.1035480

**Published:** 2022-10-21

**Authors:** Joana Ferrer-Fàbrega, Emma Folch-Puy, Andrea Llaves-López, Rocío García-Pérez, Josep Fuster

**Affiliations:** ^1^Hepatobiliopancreatic Surgery and Liver and Pancreatic Transplantation Unit, Clinic Institute of Digestive and Metabolic Diseases (ICMDiM), Hospital Clínic, University of Barcelona, Barcelona, Spain; ^2^August Pi i Sunyer Biomedical Research Institute (IDIBAPS), Barcelona, Spain; ^3^Network for Biomedical Research in Hepatic and Digestive Diseases (CIBEREHD), Barcelona, Spain; ^4^Experimental Pathology Department, Institut d'Investigacions Biomèdiques de Barcelona-Consejo Superior de Investigaciones Científicas (IIBB-CSIC), Barcelona, Spain

**Keywords:** experimental—animal models, ischemia-reperfusion injury, normothermic pancreas perfusion, organ preservation, pancreas transplantation

## Abstract

Pancreas transplantation is, at present, the only curative treatment for type-1 diabetes that maintains normoglycemia thus avoiding complications arising from poor glycemic control. Despite its great benefits, the number of pancreas transplants has decreased significantly since its inception in the late 1960s, largely due to demographic changes and the consequent suboptimal quality of donors. The selection criteria for pancreas donors mainly depend on morphological variables such as fatty infiltration, fibrosis, or edema, as well as both functional (amylase and lipase) and clinical variables of the donor. However, the final criterion in the decision-making process is the somewhat subjective assessment of a trained surgeon. That being said, the recent incorporation of graft perfusion machines into clinical practice seems to be changing the work dynamics of the donor organ retrieval team, facilitating decision-making based on objective morphological and functional criteria. Normothermic perfusion using perfusate with supplemental oxygen replicates near physiological parameters thus being a promising strategy for organ preservation. Nevertheless, optimum perfusion parameters are difficult to establish in pancreas transplantation given its complex vascular anatomy combined with an intrinsically low blood flow. The objective of this work is to analyze the results published in the recent literature relating to the considerations of *ex-vivo* normothermic graft perfusion machines and their usefulness in the field of pancreas transplantation.

## Introduction

Pancreas transplantation is currently the only treatment alternative to restore insulin independence in patients with type-1 diabetes, ameliorate the course of the secondary complications of the disease and improve both patient survival and quality of life (1). A whole pancreas may be transplanted alone, simultaneously or following an earlier kidney transplant. Simultaneous kidney-pancreas transplantation constitutes the therapy of choice for diabetic patients with end-stage renal disease, accounting for 86% of total pancreas transplant procedures (2). In addition, pancreatic islet transplantation provides a less invasive option to whole pancreas transplantation but requires multiple donor pancreases to achieve insulin independence (3).

The long-term outcomes of pancreas transplantation have significantly improved over time and the development and progression of secondary diabetic complications have been reduced or even reversed after a successful transplant (4). Despite these advances, the annual number of pancreas transplants is declining, even though the prevalence of diabetes has increased (5). At present, one of the limiting factors for whole organ pancreas and islet transplantation programs is the scarcity of suitable donors. This is due to the progressive aging of the donor population, the increasing number of obese donors, and the growing levels of donation from circulatory death (DCD) donors (6). On this basis, the “ideal” pancreas is difficult to find and transplant units have progressively broadened the standard criteria donors to include these marginal, high-risk organs. Such extended criteria donors' grafts are more vulnerable to ischemic injury and often associated with an expanded rate of early allograft dysfunction (7).

## Static cold storage of pancreas grafts: Reaching the limits of preservation in marginal donors

The organ preservation process is an essential part of transplantation to maintain function of the organ and tissue during storage. Preservation of the pancreas is critical to prevent the two major early complications of pancreas transplant, namely thrombosis and graft pancreatitis. These complications are causes of graft loss in the postoperative period and can occur in 25–50% of cases (8). The standard method to preserve human pancreas is hypothermic preservation by static cold storage (SCS). Once the organ is harvested, an immediate flush is performed with a cold preservation fluid in order to quickly cool down the organ through the elimination of remaining blood and vascular uptake of the solution. This provides an optimal environment for subsequent storage of the organ on ice in the same solution and transportation in a cooler until its implantation in the recipient. This technique is currently the standard method for pancreas preservation because of its simplicity, low costs, and favorable outcomes for high quality organs (9). However, its use is limited because of the increasing number of marginal donor organs.

During the ischemic phase of SCS, the deficiency of oxygen results in the lack of enough adenosine triphosphate (ATP) generation for continued cellular function, resulting in downstream impacts and accumulation of toxic metabolites that, in turn, exacerbate cell injury and damaging pathways after the reperfusion stage. Generation of ROS upon restoration of blood flow in the recipient promote endothelial dysfunction, and local inflammatory responses (10). Even so, although cold ischemia itself does not prevent the chemical processes that cause the ischemic injury during the preservation period, hypothermia still has a crucial role in organ preservation through the slowing down of metabolism.

Preservation solutions for SCS have also been developed to counteract ischemic injury (11). The choice of pancreas cold preservation solutions for SCS varies between countries (12).

## Moving toward *ex vivo* machine perfusion for advanced preservation and conditioning of the pancreas graft

*Ex vivo* machine perfusion (EVMP) has provided benefits over SCS in other organ transplants (8). It consists of a pump-generated flow through the organ in a circuit allowing recirculation of a preservation solution through the vasculature at various temperatures. The continuous perfusion promotes the delivery of oxygen and nutrients to the parenchyma together with the removal and “washout” of noxious metabolites. Besides improving preservation, EVMP may allow *in vivo* measurement in the perfusate of functional and biochemical parameters of the graft, providing a tool for selection and even maintenance of transplantable grafts, an especially interesting factor when using marginal grafts (13). In this context, SCS does not allow the extension of the donor pool as the decision of accepting the organ for transplantation is taken after visual macroscopic assessment, bringing us a step closer to the era of dynamic preservation strategies. Hence, the purpose of this review is to update the current knowledge of EVMP techniques with special consideration to the little explored field of *ex vivo* normothermic machine perfusion (EVNMP) for pancreas grafts. While technically more complex, the principles of EVNMP are to maintain physiological conditions. In this setting, this technology has been demonstrated to be clinically beneficial in the context of immediate graft function in organs such as the kidney, the liver, and the lung (14–16). The review will provide an overview of the fundamentals of current pancreatic EVNMP and will discuss the future use of this technique in addressing the imbalance between the current donor pancreas demand and supply by enabling the restoration of damaged pancreas and subsequently improve graft function.

The objectives set in the development of EVMP lie in the selection of useful parameters for the acceptability of the grafts and the improvement of their quality. In the field of pancreas transplantation there is still further work to be done, since some EVMP protocols cannot be directly applied to the pancreas as it is significantly more vulnerable to injury than other abdominal organs. In fact, the stricter donor selection criteria for transplantation results in a much higher discard rates of donor pancreas. The pancreas has an intrinsically low blood flow with complex vascular anatomy and is highly sensitive to ischemic injury during both organ retrieval and preservation causing a negative impact on the microcirculation of the grafts. In this sense, high perfusion pressure, such as that used in the liver and kidney EVMP, can cause acinar necrosis, edema formation and endothelial injury, which are recognized risk factors for early graft pancreatitis and thrombosis. On the contrary, if the pressure is too low, perfusate blood flow will not be appropriate for an effective perfusion of the microvasculature and an adequate oxygenation and nutrient supply (17).

As detailed below, hypothermic and normothermic perfusion machines have currently emerged as innovative procedures for advanced organ preservation and conditioning in clinical practice.

### *Ex vivo* hypothermic machine perfusion

*Ex vivo* hypothermic machine perfusion (EVHMP) implies the recirculation of cooled preservation solution through the vascular system of the organ in either a continuous or a pulsatile flow. The maintenance of the donor organ on a device at low temperatures is meant to prevent ATP depletion reducing the deleterious effects of ischemia and reperfusion injury. This is one of the advantages that have made EVHMP become an alternative to SCS, particularly in kidney and liver preservation (18, 19). Another advantage is that the temperature can be accurately regulated to rest in the preferable range of 4–8°C. In SCS on ice, temperatures frequently fall around or below 0°C, inducing crystal formation within the cytoplasm and thus resulting in more severe tissue injury upon reperfusion (20). Furthermore, compared to SCS, EVHMP can expand the donor pool by enabling the surgeon to accept marginal donors and those with longer preservation times. On the contrary, it does not allow the functional assessment of the graft under hypothermic conditions (21).

To date, there are no publications reporting clinical pancreas transplant using EVHMP strategies and most studies have been undertaken using animal models or discarded human pancreata. All these pre-clinical studies have been performed at low perfusion pressures thus indicating low pressures as a safe condition for pancreas EVHMP. However, some limitations of these studies are that they are mainly based on histological findings and the lack of post-transplant evaluation. In these cases, a normothermic reperfusion could be used to mimic the physiological environment of transplantation and allow an optimal assessment of organ viability.

### *Ex vivo* normothermic machine perfusion

*Ex vivo* normothermic machine perfusion (EVNMP) has been developed as a resuscitation tool for the organ after a period of SCS and is employed for short space of time i.e., while the recipient is being prepared for transplant. Subsequently, the graft is provisionally returned to SCS until transplantation (22).

Compared to EVHMP, EVNMP allows a complete functional assessment of the graft under physiologic conditions, usually between 34°C and 39°C. This fact makes EVNMP an invaluable tool for *ex vivo* functional analysis as well as the technique can also be applied to extend the storage periods, which may help facilitate long-distance transportation. Another unique advantage is the ability to pretreat the organ to deliver therapeutic agents *ex vivo* prior to transplantation in the recipient (23).

Normothermic organ preservation demands a physiological environment with appropriate oxygen, nutrition, and metabolic substrates to replace consumed cellular energy resources. Additionally, to reduce oxidative damage, the perfusate solution needs to stabilize electrolyte balance and cell fluid content to decrease edema and reduce free radical peroxide scavengers (24). In clinical practice, the most used perfusate solution contains a red cell-based solution enriched with nutrients and also containing physiological buffers and supplementary constituents. Normothermic storage with whole blood cells-based solutions have been also investigated, although it has been demonstrated that the blood may contain different factors, such as antibodies, clotting factors, activated leukocytes and thrombocytes, which may aggravate ischemia reperfusion injury. During this process, inflammatory mediators are generated together with the activation of complement cascades (25). Subsequently, leukocyte-depleted blood and plasma-free based perfusates have been well-established in both pre-clinical and clinical studies.

Hosgood et al. were the first to use EVNMP to assess kidney viability pre-transplantation. In this study, a short period of normothermic perfusion was applied to a discarded kidney before its transplantation (26). Then, in a small clinical trial, 1 hour of normothermic oxygenated perfusion permitted resuscitation and evaluation of 10 declined DCD kidneys using a quality score based on gross appearance, renal blood flow and total urine output (14). Meanwhile, recent results from a clinical trial on 31 discarded livers demonstrated the feasibility of EVNMP and the successful transplantation of 71% of perfused livers without any incidence of primary non-function in addition to a 100% 90-day patient and graft survival (27).

## *Ex vivo* normothermic machine perfusion parameters to enable pancreas graft quality and viability

In the field of pancreas transplantation, normothermic preservation technologies still await a breakthrough. A number of studies have been reported, either experimental or pre-clinical, and the vast majority used a deficient number of pancreas and are mainly based on histological findings and poor biochemical analysis. Because of this, there is a need to establish the optimal assessment parameters to determine graft quality and viability.

The first investigation of normothermic perfusion in the pancreas was reported in 1926 by Babkin and Starling using isolated dog pancreas (28). Other systems were subsequently developed for different animal models to assess viability and function after various durations and storage conditions (29–44). It has not been until the last 10 years (Tables 1, 2) that interest in the subject has been reactivated owing to the critical shortage in organ donation and the fact that a greater number of extended criteria donors are used for transplantation. In 2017, the first study in porcine pancreas established a model of perfusion in an oxygenated roller pump with and without addition of the kidney to the DBD pancreas as a dialysis organ (45). The perfusate consisted of autologous whole blood incorporating cephazolin, epoprostenol sodium, and heparin. Despite minimal cold ischemia time (CIT), stable perfusion and maintenance of acid-base homeostasis, exocrine pancreatic tissue displayed acinar damage, inflammation, and thrombosis while islets cells remained relatively spared. The explanation of the issues highlighted by the EVNMP groups was that, as the perfusion solution is continuously recycled around the circuit, the levels of proteolytic enzymes produced by the pancreas increase progressively throughout perfusion. The use of whole blood as perfusate must also be taken into consideration as it could potentially exacerbate inflammatory response and tissue damage. Likewise, perfusion pressure in this study was 70–80 mmHg, which could be considered too high for the pancreatic organ and thus a contributor to the acinar injury.

**Table 1:** Experimental EVNMP studies in animal models.

						**Perfusion characteristics**				**Arterial parameters**			**Studies and analysis**			
**Author** **[ref] (Year)**	**Model** **(strain; weight) *n***	**Donor model (DBD/DCD)**	**Mean pancreatic weight**	**CIT**	**Perfusion device** **(pulsatile/** **non-pulsatile)**	**Perfusion time**	**Perfusate (volume)** **(protease inhibitor; colloid)**	**Dialysis circuit**	**% Oxygen**	**Arterial pressure**	**Arterial bloodflow rate**	**Samples**	**Histopathological analysis**	**Blood gas analysis**	**Biochemical analysis**	**Transplant**
Kuan (45) (2017)	Pig (domestic white; 40-50kg) *n =* 4 (2 pancreases alone, 2 pancreas + kidney)	DBD	NS	34 ± 7.78 min	SARNS 8000 extracorporeal roller pump (3M, St. Paul, MN, USA), Baby-RX venous reservoir and membrane oxygenator (Terumo, Ann Arbor, MI, USA), and water bath temperature regulator (pulsatile)	4 h for pancreas alone, 2 h for pancreas + kidney	Autologous whole blood, cephazolin, epoprostenol sodium, heparin (1.8–2 L) (no; no)	Yes (addition of the porcine kidney in 2 of the animals)	NS	70–80 mmHg for pancreas alone, 90–100 mmHg for pancreas + kidney	200 ml/min	Perfusate, blood, pancreas biopsies and urine	Gross appearance Hematoxylin and eosin stain	Yes	Biochemical analysis of blood samples not performed due to haemolysis Perfusion dynamics Endocrine function under glucose stimulation	No
Kumar (46) (2018)	Pig [Yorkshire landrace; 45-55 kg) *n =* 13 (9 pancreases control group (50mmHg), 4 pancreases low-pressure group (20 mmHg)]	DCD	NS	127 min (control group), 136 min (low pressure group)	Affinity CP Centrifugal pump and Minimax Plus oxygenation System (Medtronic Inc., (Minneapolis, MN, USA) with a thermostatic water-based heat exchanger unit (non-pulsatile)	2–4 h (control group), 4 h (low pressure group)	Autologous whole blood, cefuroxime, epoprostenol sodium (1.5 L) (no; no)	NS	NS	50 (control group; high), 20 (low pressure group). From other studies the group stablished that 50 mmHg of pressure was optimal to prevent edema in the graft.	141 ml/min (control group), 40 ml/min (low-pressure group)	Perfusate, pancreatic juice, blood and pancreas biopsies	Hematoxylin and eosin stain Immunohistochemistry (caspase-3, M30 cytodeath and ATP synthetase)	Yes	Exocrine function in perfusate through amylase and lipase levels and production of pancreatic juice Endocrine function under glucose stimulation Measure of hemoglobin, lactate, glucose, potassium, sodium and chloride	No
Hamaoui (47) (2018)	Pig (NS; NS) *n =* 10	DCD	NS	3–7 h	Waters Medical RM3 Machine Perfusion Unit with oxygenator (Waters Medical Systems, Rochester, MN) (non-pulsatile)	2 h	Autologous whole blood:saline mixture (3:1) supplemented with NaH2CO3 (NS) (no; no)	NS	95%	45 mmHg	40–70 ml/min/100 g	Pancreatic exocrine and duodenal secretions, pancreas biopsies	Hematoxylin and eosin stain	Yes	Exocrine function in pancreatic exocrine and duodenal secretions through amylase Endocrine function (insulin levels) under glucose stimulation Levels of tissue lactate	No
Ogbemudia (48) (2021)	Pig (domestic; 50–70 kg) *n =* 13 (4 pancreases 6 h SCS + 1 h EVNMP; 4 pancreases 6 h UW-EVHMPO + 1 h EVNMP; 5 pancreases 6 h IGL2-EVHMPO + 1 h EVNMP)	DCD	NS	9h (3h + 6h)	Deltastream DP3 diagonal pump (MEDOS Medizintechnik AG, Stolberg, Germany), oxygenator and heat exchanger unit (MEDOS HILITE 2800 LT TM) (pulsatile)	1 h	Autologous red blood cells and plasma without leukocytes, coamoxiclav, heparin (800 ml) (no; no)	NS	95%	40 ± 8 mmHg	67 ± 43 ml/min (IGL2-EVHMPO group), 137 ± 86 ml/min (UW-EVHMPO group), 46 ± 32 ml/min (SCS group)	Perfusate, pancreas biopsies	Gross appearance	Yes	Water mass in graft samples Lipase, amylase, lactate, and lactate dehydrogenase (LDH) in perfusate Endocrine function under glucose stimulation	No
Mazilescu (49) (2022)	Pig [Yorkshire; 30 kg (7 pigs, EVNMP), 40 kg (3 pigs EVNMP+Tx)] *n =* 10 (7 pancreases for 6 h EVNMP, 3 pancreases for 3 h EVNMP+Tx)	NS	143.9 ± 17.6 g after retrieval, 201.4 ± 32.1 g after perfusion	NS	S3 heart-lung machine and neonatal cardiopulmonary bypass equipment consisting of a centrifugal pump, an oxygenator (Sorin Group Inc., Markham, Canada) with an addition of heat exchanger (non-pulsatile)	6 h EVNMP, or 3 h EVNMP +Tx	Ringer's lactate, STEEN Solution (XVIVO Perfusion AB, Goteborg, Sweden), washed leukocyte-filtered erythrocytes, double reverse osmosis water, sodium bicarbonate, calcium gluconate, heparin, aprotinin (NS) (aprotinin, albumin)	Yes	95%	25 mmHg	120 ± 21 ml/min initially, 101 ± 15 ml/min toward end of perfusion	Perfusate, pancreatic juice, duodenal samples and pancreas biopsies	Hematoxylin and eosin stain	Yes	LDH, Glucose, insulin, C-peptide and bicarbonate concentration Inflammatory cytokines Mild hemolysis but possible assessment of amylase Endocrine function under glucose stimulation	Yes (*n =* 3), diabetes was induced by pancreatectomy in recipients prior to Tx

DBD, Donation after Brain Death; DCD, Donation after Circulatory Death; CIT, Cold Ischemia Time; Tx, Transplantation; NS, Not Specified; EVNMP, *Ex Vivo* Normothermic Machine Perfusion; EVHMPO, *Ex Vivo* Oxygenated Hypothermic Machine Perfusion.

**Table 2:** Experimental EVNMP studies in discarded human pancreata.

				**Perfusion characteristics**				**Arterial parameters**			**Studies and analysis**		
**Author [ref] (Year)**	**Donor type** **(DBD/DCD) *n***	**CIT**	**Perfusion device** **(pulsatile/non-pulsatile)**	**Perfusion time**	**Perfusate (volume) (protease inhibitor; colloid)**	**Dialysis circuit**	**% Oxygen**	**Arterial pressure**	**Arterial bloodflow rate**	**Samples**	**Histopathological analysis**	**Blood gas analysis**	**Biochemical analysis**
Barlow (50) (2015)	3 DBD + 1DCD *n =* 5 (1 excluded due to elevated CIT)	13–18 h	Customized pediatric cardiopulmonary bypass technology developed for kidney EVNMP (Medtronic Inc.) with an organ chamber, venous reservoir, Biopump 560 centrifugal blood pump, oxygenator, and a heat exchanger (Chalice Medical Ltd, United Kingdom) (non-pulsatile)	1–2 h	Blood-based perfusate mixture (donor ABO-compatible red blood cells) diluted with Gelofusine, additives (sodium bicarbonate, mannitol, glucose, heparin) (NS) (NS; Gelofusine)	NS	95 %	50–55 mmHg	35 ± 2.8 mL/min/100 g	Perfusate, pancreas biopsies	Gross appearance Hematoxylin and eosin stain in biopsies	Yes	Exocrine function in perfusate through amylase and lipase levels Endocrine function (insulin levels) under glucose stimulation
Hamaoui (47) (2018)	NS *n =* 3 (1 excluded due to massive edema)	26.8 and 56 h respectively	Waters Medical RM3 Machine Perfusion Unit with oxygenator, heat exchanger and pump head (Waters Medical Systems, Rochester, MN) (non-pulsatile)	2 h	Krebs-Henseleit buffer-based solution, NaH_2_CO_3_ (NS) (no; no)	NS	95 %	40 and 32 mmHg	32.2 and 49.8 mL/min/100 g, respectively	Pancreatic exocrine and duodenal secretions, pancreas biopsies	Gross appearance Hematoxylin and eosin stain in biopsies	NS	Exocrine function in perfusate through amylase levels Endocrine function (insulin levels) under glucose stimulation
Nassar (51) (2018)	DBD *n =* 3	4 h	Customized liver perfusion device. Centrifugal pump, oxygenator, and heater exchanger (Medtronic Inc.) with an added Sarns 8000 Roller Pump (Terumo, Ann Arbor, MI, USA) (non-pulsatile)	Two grafts perfused for 6 h, one for 12 h	Packed red blood cells and plasma 1:3 ratio (NS) (NS; NS)	NS	NS	60 mmHg	55 mL/min/100 g	Perfusate, pancreas and duodenal secretions, pancreas biopsies	Chromogranin A stain of pancreatic exocrine parenchyma Hematoxylin and eosin stain of pancreas acinus and islets	NS	Exocrine biochemical evaluation trough bicarbonate levels in the duodenum C-peptide levels

DBD, Donation after Brain Death; DCD, Donation after Circulatory Death; CIT, Cold Ischemia Time; NS, Not Specified; EVNMP, *Ex Vivo* Normothermic Machine Perfusion.

The same group directly studied the effects of EVNMP testing two different pressure perfusions in a porcine DCD pancreata model (46). In this study, an oxygenated non-pulsatile pump was used with a whole blood-based perfusate to which heparin was added to reduce coagulation. The control group was perfused at 50 mmHg and was compared to an experimental group of “low pressure” grafts perfused at 20 mmHg. Both pancreatic blood flow and pressure remained stable, but control grafts achieved a mean blood flow of 140 ml/min, while the low-pressure group achieved a blood flow of only 40 ml/min, indicating a worse perfusion. All grafts showed evidence of oxygen absorption and cellular viability, as corroborated with immunohistochemistry. Both endocrine and exocrine functionality were preserved, but amylase levels were significantly lower at all time points in the low-pressure group compared with controls. Notwithstanding, cell viability and ATP synthetase stain showed an improved score grade in the low-pressure group. This is the only study on EVNMP in porcine pancreas that measures ATPase activity, a marker of viability which has been related to better transplantation outcome (52). Despite some controversy in the results, the authors suggest low pressure perfusion as an improved method for graft preservation during EVNMP.

Hamaoui et al. (47) and Ogbemudia et al. (48) developed a normothermic hemoperfusion circuit for the pancreas used in parallel to the design of the EVHMP porcine model with the aim of studying the feasibility assessment of the graft. However, as this is a different concept than the application of “pure” *ex vivo* normothermia for reversing ischemia injury, these results should be carefully analyzed.

A recent study has demonstrated the safety of pancreas transplantation after EVNMP in a porcine model using an oxygenated non-pulsatile pump in combination with a dialysis circuit (49). The perfusate consisted of STEEN solution. The solution included human serum albumin which provides an optimal colloid osmotic pressure and dextran to preserve the endothelium from excessive leukocyte interaction. In addition, washed-leukocytes-filtered erythrocytes, sodium bicarbonate, heparin and aprotinin were added. Seven out of ten pancreas were subjected to 6 h of EVNMP. The grafts showed stable perfusion parameters, active metabolism, homeostasis, and only mild graft injury assessed through histology. Initially, arterial blood flow rate was around 120 ml/min. and slightly decreased to 101 ml/min, likewise maintaining high levels which allow for a better perfusion of the graft. Cytokine release during EVNMP in the perfusate showed an increase in IL-6 pro-inflammatory and TGF-β1 and TGF-β2 anti-inflammatory cytokines although high levels of cytokines were found in the dialysate, which probably served to minimize the inflammation and graft injury during EVNMP. In this study, glucagon stain was used to highlight preserved islets cells. To demonstrate the reliability of the perfusion system, the remaining three pancreases were subjected to 3 h of perfusion prior to transplantation and the recipients were then observed for 48–72 h post-transplantation. EVNMP grafts had normal function based on histological findings and maintained physiologic glucose levels. In spite of these promising results, further studies need to be performed for a more complete and accurate assessment of the graft function after transplantation.

Finally, EVNMP of discarded human pancreata has been successfully demonstrated relatively recently by two groups despite none of the organs being either transplanted or undergoing islet isolation protocol. Barlow et al. adapted their kidney EVNMP technique to initiate EVNMP of four non-transplantable pancreases (50). A red blood cell-based perfusate mixture was diluted with Gelofusine as colloid plus additives (sodium bicarbonate, mannitol, glucose, and heparin). All organs demonstrated adequate perfusion, pancreatic exocrine function, and insulin production. Histopathology demonstrated varying degrees of damage, reflective of the heterogeneous features of pancreas donor, ranging from focal to extended acinar necrosis. Arterial pressure was maintained at 50-55 mmHg, which could be considered high. Furthermore, the incorporation of a dialysis unit to the system or the addition of antiproteases to the perfusate are two potential factors that could help reduce pancreas injury.

The translational study published by Hamaoui et al. (47) applied the protocols developed in porcine models to three humans' pancreata. As mentioned before, the EVNMP circuit was designed for functional assessment of the pancreas after maintaining the graft in hypothermic conditions, i.e, its applicability is therefore limited.

Nassar et al. (51) utilized a modified liver perfusion machine for three DBD donor human pancreases. Perfusion times were longer (6–12 h) compared to Barlow et al. (1–2 h) but CIT was much lower (4 vs. 13 h in the study of Barlow et al.). Results revealed endocrine function at both 6- and 12-h through the determination of C-peptide production and by chromogranin staining. At the end of perfusion, histology showed well-preserved cell architecture without necrosis or hemorrhage in 2 out of 3 pancreases. Taken together, both studies show that EVNMP of discarded human donor pancreata is potentially achievable and allows cell injury and functional evaluation.

Despite the difficulty of making firm conclusions from the research findings on pancreas EVNMP, which are based strictly on published data, it seems at this point feasible to recommend the use of lower arterial pressure to avoid endothelial injury while achieving higher flow rates to allow a better perfusion.

From the studies, we have also concluded that perfusate-containing packed red blood cells improve outcomes when compared to whole blood perfusate. Thus, a red blood cell-based perfusate solution with adequate oxygenation (95%) and supplemented with antiprotease, colloid, antibiotics and heparin may provide a prolonged preservation of the pancreas grafts. More studies are needed to assess the usefulness of other red blood cell-based perfusates combined with artificial acellular solutions.

Regarding the time of perfusion, it is challenging to set a range but one may consider that, when using longer periods of perfusion, the use of a dialysis circuit may help to reduce edema formation and recirculation of metabolic toxins.

Nevertheless, to date, and with these limited publications in pancreas EVNMP, there are no clear, approved, and accurate conditions to enable its implementation on a routine basis in pancreatic clinical settings. Significant refinements to the technique and rigorous analysis of data from larger studies are urgently required. Obviously, we would also need further perfusion studies with transplanted grafts to corroborate that the promising findings related to the suitability of the EVNMP system can be reproduced in a clinical model.

## Conclusion

Although the number of extended criteria donor organ transplantation has increased, there has been minor progression in the development of a proper quality assessment for these organs. At present, the EVNMP technique allows organ monitoring and estimation of its quality before transplantation, unlike clinical pancreas transplantation, for which there are few experimental and pre-clinical studies published.

Outcome measures in current studies of EVNMP-treated pancreas include blood flow, blood gas analysis (acid-base homeostasis, pH, bicarbonate, and oxygen consumption), mean arterial pressure, endocrine function under glucose stimulation, amylase and lipase levels, production of pancreatic secretion, metabolic markers, and histologic analysis. There is still disagreement concerning the optimal parameters such as the set of perfusion pressures or flow rates to enable an optimal post-transplant pancreatic graft function. In that vein, the lack of accurate pre-clinical methodological design may lead to errors in the analysis of results, affecting the quality of the research and thus contribute to the frequent scarcity of reliable animal studies.

## Data availability statement

The original contributions presented in the study are included in the article/supplementary materials, further inquiries can be directed to the corresponding author.

## Author contributions

Study conception and design: JF-F and JF. Data acquisition, analysis, and interpretation: JF-F, EF-P, AL-L, and RG-P. Drafting of manuscript and critical revision of manuscript: JF-F, EF-P, AL-L, RG-P, and JF. All authors agree to be accountable for the content of the work. All authors contributed to the article and approved the submitted version.

## Funding

This study has been supported by Instituto de Salud Carlos III (ISCIII) through the Project PI18/00161 and co-funded by the European Union awarded to JF-F. This work has been supported by grant from the Ministerio de Ciencia, Innovación y Universidades, reference PID2019-104130RB-I00 awarded to EF-P. AL-L was recipient of a Grant (Ayudas para contratos predoctorales para la formación de doctores convocatoria 2020; Reference PRE2020-096502) funded by the Ministerio de Ciencia e Innovación (MICINN).

## Conflict of interest

The authors declare that the research was conducted in the absence of any commercial or financial relationships that could be construed as a potential conflict of interest.

## Publisher's note

All claims expressed in this article are solely those of the authors and do not necessarily represent those of their affiliated organizations, or those of the publisher, the editors and the reviewers. Any product that may be evaluated in this article, or claim that may be made by its manufacturer, is not guaranteed or endorsed by the publisher.
